# Prevalence and Significance of the Vessel-Cluster Sign on Susceptibility-Weighted Imaging in Patients With Severe Small Vessel Disease

**DOI:** 10.1212/WNL.0000000000200614

**Published:** 2022-08-02

**Authors:** Salvatore Rudilosso, Ernest Chui, Michael S. Stringer, Michael Thrippleton, Francesca Chappell, Gordon W. Blair, Daniela Jaime Garcia, Fergus Doubal, Iona Hamilton, Anna Kopczak, Michael Ingrisch, Danielle Kerkhofs, Walter H. Backes, Julie Staals, Robert van Oostenbrugge, Marco Duering, Martin Dichgans, Joanna M. Wardlaw, on behalf of the SVDs@Target Investigators

**Affiliations:** From the Comprehensive Stroke Center (S.R.), Department of Neuroscience, Hospital Clinic, University of Barcelona; August Pi i Sunyer Biomedical Research Institute (IDIBAPS)(S.R.), Barcelona, Spain; Centre for Clinical Brain Sciences (E.C., M.S.S., M.T., F.C., G.B., D.J.G., F.D., I.H., J.M.W.), UK Dementia Research Institute, University of Edinburgh, United Kingdom; Institute for Stroke and Dementia Research (A.K., M. Dichgans), University Hospital, LMU Munich; Department of Radiology (M.I.),Ludwig-Maximilians-University Hospital Munich, Germany; Department of Neurology (D.K., J.S., R.v.O.), CARIM—School for Cardiovascular Diseases Maastricht University Medical Center+, Maastricht,; Department of Radiology & Nuclear Medicine (W.H.B.), School for Mental Health & Neuroscience and School for Cardiovascular Diseases, Maastricht University Medical Centre, Netherlands; Institute for Stroke and Dementia Research (ISD) (M. Duering), University Hospital, LMU Munich, Germany; Medical Image Analysis Center (MIAC AG) and Department of Biomedical Engineering (M. Duering), University of Basel, Switzerland; Munich Cluster for Systems Neurology (SyNergy) (M. Dichgans); and German Center for Neurodegenerative Diseases (DZNE) (M. Dichgans), Munich, Germany.

## Abstract

**Background and Objectives:**

Magnetic resonance susceptibility-weighted imaging (SWI) can identify small brain blood vessels that contain deoxygenated blood due to its induced magnetic field disturbance. We observed focal clusters of possible dilated small vessels on SWI in white matter in severe small vessel disease (SVD). We assessed their prevalence, associations with SVD lesions, and vascular reactivity in patients with sporadic SVD and in patients with cerebral autosomal dominant arteriopathy with subcortical infarcts and leukoencephalopathy (CADASIL).

**Methods:**

Secondary cross-sectional analysis of a prospective multicenter observational study of patients with either sporadic SVD or CADASIL (INVESTIGATE-SVD) studied with 3 Tesla MRI including blood-oxygen-level-dependent MRI cerebrovascular reactivity (CVR). Two independent raters evaluated SWI sequences to identify “vessel-clusters” in white matter as focal low-signal dots/lines with small vessel appearance (interrater agreement, kappa statistic = 0.66). We assessed per-patient and per-cluster associations with SVD lesion type and severity on structural MRI sequences. We also assessed CVR within and at 2-voxel concentric intervals around the vessel-clusters using contralateral volumes as a reference.

**Results:**

Among the 77 patients enrolled, 76 had usable SWI sequences, 45 with sporadic SVD (mean age 64 years [SD 11], 26 men [58%]) and 31 with CADASIL (53 years [11], 15 men [48%]). We identified 94 vessel-clusters in 36 of the 76 patients (15/45 sporadic SVD, 21/31 CADASIL). In covariate-adjusted analysis, patients with vessel-clusters had more lacunes (OR, 95% CI) (1.30, 1.05–1.62), higher white matter hyperintensity (WMH) volume (per-log10 increase, 1.92, 1.04–3.56), and lower CVR in normal appearing white matter (per %/mm Hg, 0.77, 0.60–0.99), compared with patients without vessel-clusters. Fifty-seven of the 94 vessel-clusters (61%) corresponded to noncavitated or partially cavitated WMH on fluid-attenuated inversion recovery, and 37 of 94 (39%) to complete cavities. CVR magnitude was lower than in the corresponding contralateral volumes (mean difference [SD], *t*, *p*) within vessel-cluster volumes (−0.00046 [0.00088], −3.021, 0.005) and in the surrounding volume expansion shells up to 4 voxels (−0.00011 [0.00031], −2.140, 0.039; −0.00010 [0.00027], −2.295, 0.028) in vessel-clusters with complete cavities, but not in vessel-clusters without complete cavitation.

**Discussion:**

Vessel-clusters might correspond to maximally dilated vessels in white matter that are approaching complete tissue injury and cavitation. The pathophysiologic significance of this new feature warrants further longitudinal investigation.

Cerebral small vessel disease (SVD) refers to a spectrum of ischemic and hemorrhagic brain lesions resulting from heterogenous pathophysiologic processes involving perforating arterioles, capillaries, and venules.^1^ Recent small subcortical infarcts, lacunes, white matter hyperintensities (WMH), enlarged perivascular spaces (PVS), and cerebral microbleeds are the typical SVD features on standard structural MRI protocols that may be observed in both sporadic and genetic SVD forms.^2^ Other structural, hemodynamic, and functional features of SVD may be assessed using advanced imaging techniques, revealing possible mechanisms of SVD including disordered vasoreactivity.^3^

Susceptibility-weighted imaging (SWI) is a gradient-echo–derived MRI sequence that is commonly used to identify hemorrhagic features in SVD (i.e., microbleeds),^4^ but may also enable the study of medullary venules in the white matter,^5^ by exploiting the paramagnetism of deoxygenated blood in veins. Recent work suggested that the decreased number and altered morphology of deep medullary veins may be related to WMH load.^5-8^ On inspection of high-quality MRI scans from patients with severe SVD, we recently observed small clusters of linear-like structures in deep WMH with different grades of cavitation that showed similar signal characteristics to the medullary veins on SWI (Figure 1) but did not follow their distribution (eFigure 1, links.lww.com/WNL/C29). We hypothesized that these may represent grouped small dilated vessels associated with white matter injury and cavitation (i.e., lacunes). In this study, we describe the prevalence and characteristics of these possible clusters of small dilated vessels on SWI, their associations with patient demographics, SVD lesions, and measures of vascular reactivity, in patients with sporadic SVD or with monogenetic SVD (cerebral autosomal dominant arteriopathy with subcortical infarcts and leukoencephalopathy [CADASIL]).

**Figure 1:**
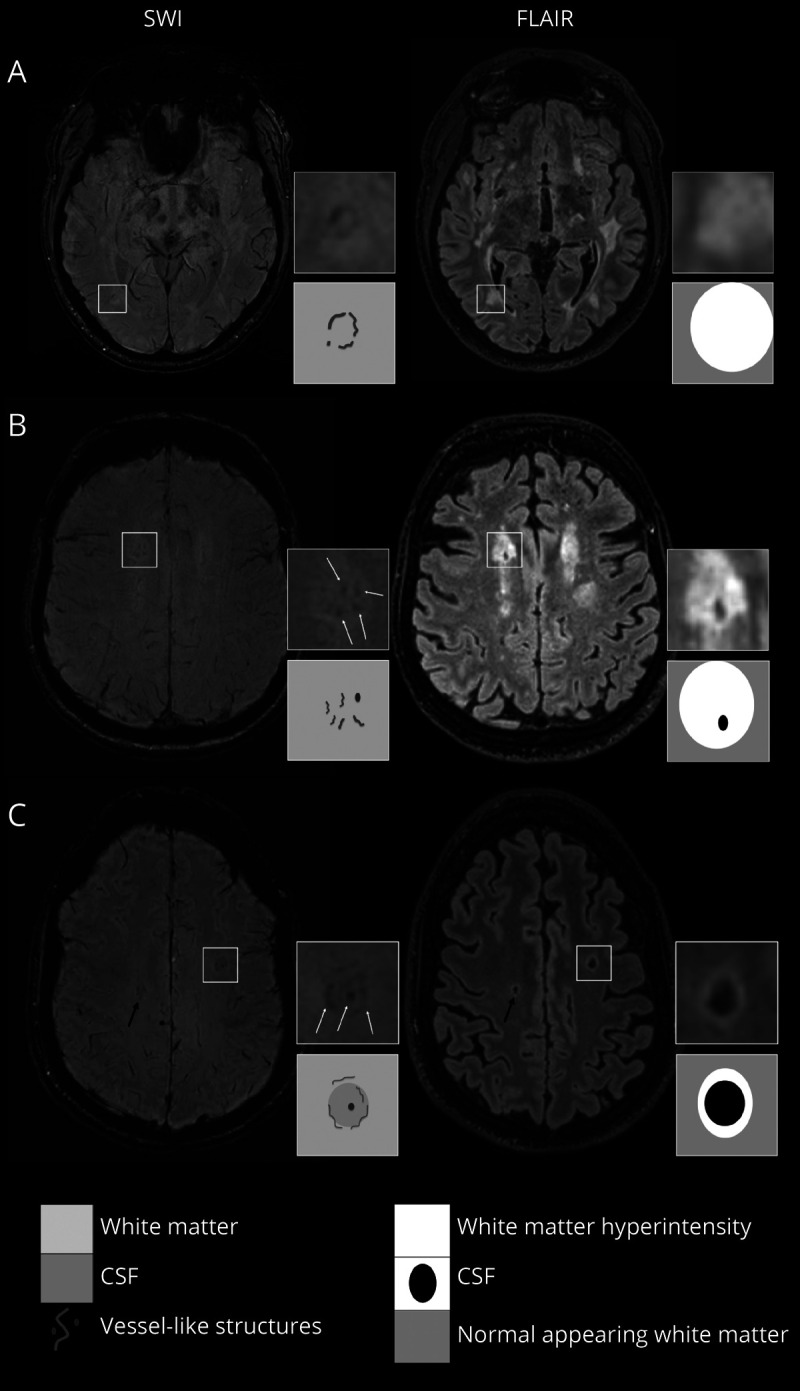
Vessel-Clusters on Susceptibility-Weighted Imaging and Corresponding Appearance on Fluid-Attenuated Inversion Recovery Vessel-clusters indicated in squares have been augmented (4x) to show details of susceptibility-weighted imaging (SWI) and fluid-attenuated inversion recovery (FLAIR) sequences, another not augmented vessel-cluster is indicated with a black arrow (C). For each patient (A with CADASIL, B and C with sporadic SVD), we present the most informative SWI (left) and corresponding FLAIR (right) sequences to show vessel-cluster appearance. Vessel-clusters are characterized by clustered small tubular structures, visible as low-signal dots (B and C) or lines (A, B, and C) on SWI not corresponding to normal appearing deep medullary veins (visible as mild low-signal parallel lines reaching the lateral ventricles from the deep white matter). Vessel-clusters are seen within white matter hyperintensities with different grades of cavitation: none (A), partial (B), or complete (C) cavitation. The schematic appearance of vessel-clusters on SWI and grade of cavitation on FLAIR are represented in the corresponding squares below the augmentations. SVD = small vessel disease.

## Methods

### Standard Protocol Approvals, Registrations, and Patient Consents

This was a secondary cross-sectional analysis from a prospective multicenter observational study (Imaging NeuroVascular, Endothelial and STructural Integrity in prepAration to TrEat Small Vessel Diseases, INVESTIGATE-SVD, ISRCTN 10514229).^9^ The study was approved by the local ethics committee in each participating institution, and informed consent was obtained from all participants. The full protocol of the study is available elsewhere.^9^

### Patients

The INVESTIGATE-SVD study cohort included patients with symptomatic sporadic (a lacunar ischemic stroke in the past 5 years or vascular cognitive impairment with SVD) or genetic SVD (diagnosis of CADASIL).^9^ Patients with other causes of stroke such as ≥50% luminal stenosis, major-risk cardioembolic source of embolism (i.e., atrial fibrillation), and other specific causes of stroke identified (i.e., hemorrhage and arteritis) were not enrolled in the study.^9^ The patients with sporadic SVD were recruited from centers in Edinburgh (UK) and Maastricht (the Netherlands), and patients with CADASIL from Munich (Germany). We excluded patients with unusable SWI acquisition. We collected a full medical history, including demographic factors (age and sex), vascular risk factors (history of hypertension, diabetes mellitus, hyperlipidemia, current smoking habit), alcohol intake, history of ischemic heart disease and peripheral vascular disease, previous stroke, prescribed statins, and antihypertensive and antithrombotic treatment. Systolic blood pressure and diastolic blood pressure were recorded at MRI visit.

### Image Acquisition

The full image acquisition protocol has been previously published.^9^ In brief, images were acquired using 3 Tesla MRI scanners (all Siemens Prisma, Siemens Healthineers) in all 3 centers using a standardized protocol, within 3 months after enrollment (median 7 days, interquartile range [IQR] 1–41). The study protocol included T1-weighted, T2-weighted, fluid-attenuated inversion recovery (FLAIR), and SWI sequences. Cerebrovascular reactivity (CVR) was assessed using an established validated blood-oxygen-level-dependent (BOLD) MRI with CO_2_ challenge.^9^

### Image Qualitative Analysis

Radiologic markers of SVD, that is, lacunes, PVS, microbleeds, and WMH, on structural MRI sequences were identified according to STRIVE criteria^2^ and graded using validated qualitative scales for WMH (Fazekas scale)^10^ and PVS load scale in basal ganglia and centrum semiovale.^11^ All image analysis was centralized and conducted by an analyst not involved in the clinical assessments and masked to patient characteristics and CVR results and performed before the assessment of this study.^9^

We defined as “vessel-clusters” the presence on SWI of grouped low-signal small tubular-like structures (seen as dots or lines depending on the orientation in reference to the axial plane) in white matter (Figure 1) that appear like small vessels but present a disorganized distribution compared with the large deep medullary veins with normal appearance on SWI (eFigure 1, links.lww.com/WNL/C29).^12^ Hence, the definition of “vessel-cluster” is based on its radiologic appearance, although the nature of such finding may need further confirmation in pathology studies.

An experienced stroke neurologist (S.R.) and a trained medical student (E.C.) independently evaluated the SWI acquisitions to identify vessel-clusters and the number of vascular-like structures per cluster (maximum intensity projection [MIP] sequences were also checked to track tubular vessel-like structures). Disagreements were resolved by discussion with a third expert neuroradiologist (J.M.W.). Interrater agreement was substantial for both the presence of the clusters (kappa statistic = 0.66, 95% CI 0.49–0.84, *p* < 0.001) and the number of single–vessel-like structures in each cluster (quadratic kappa statistic = 0.64, 95% CI 0.52–0.75, *p* < 0.001). Details of visual identification of vessel-clusters and interobserver reliability analysis are available in the Supplemental material (eMethods, eFigure 2 and eFigure 3, links.lww.com/WNL/C29).

We described the location of the vessel-clusters referring to the spatial relationship with anterior and posterior horns of the lateral ventricles (anterior, middle, and posterior white matter), as previously described,^13^ and whether the vessel-like structures appeared as a linear rim at the edges of a cavitation in the white matter. The volume (mL) and shape (round, ovoid, linear, or irregular) of the regions of the brain covered by each vessel-cluster were assessed after segmentation of the regions of interest (eMethods, links.lww.com/WNL/C29). The features of the brain areas corresponding to the vessel-clusters were assessed on structural sequences (FLAIR, T1w, and T2w) and classified on the basis of previous work^14^: normal appearing white matter, noncavitated WMH, partially cavitated WMH (either lacy appearance or incomplete cavities linked by residual strands of tissue), and completely cavitated lesions containing CSF-like liquid. Some examples of clusters and related tissue appearance on structural sequences are shown in Figure 1.

### Image Quantitative Analysis

Normal appearing white matter and WMH were segmented using a validated semiautomated pipeline.^15^ We obtained intracranial, normal appearing white matter, WMH volumes, and calculated standardized WMH volume (WMH volume/intracranial volume). For the analysis of CVR magnitude and delay within normal appearing white matter and WMH, we eroded the outer margin of the original white matter mask by 2.5 mm (1 voxel) to reduce the influence of partial volume effects. In addition, we masked the images with a dilated ventricle mask to exclude contamination from ventricular CSF and normal vessels running along the ventricle walls.

**Figure 2:**
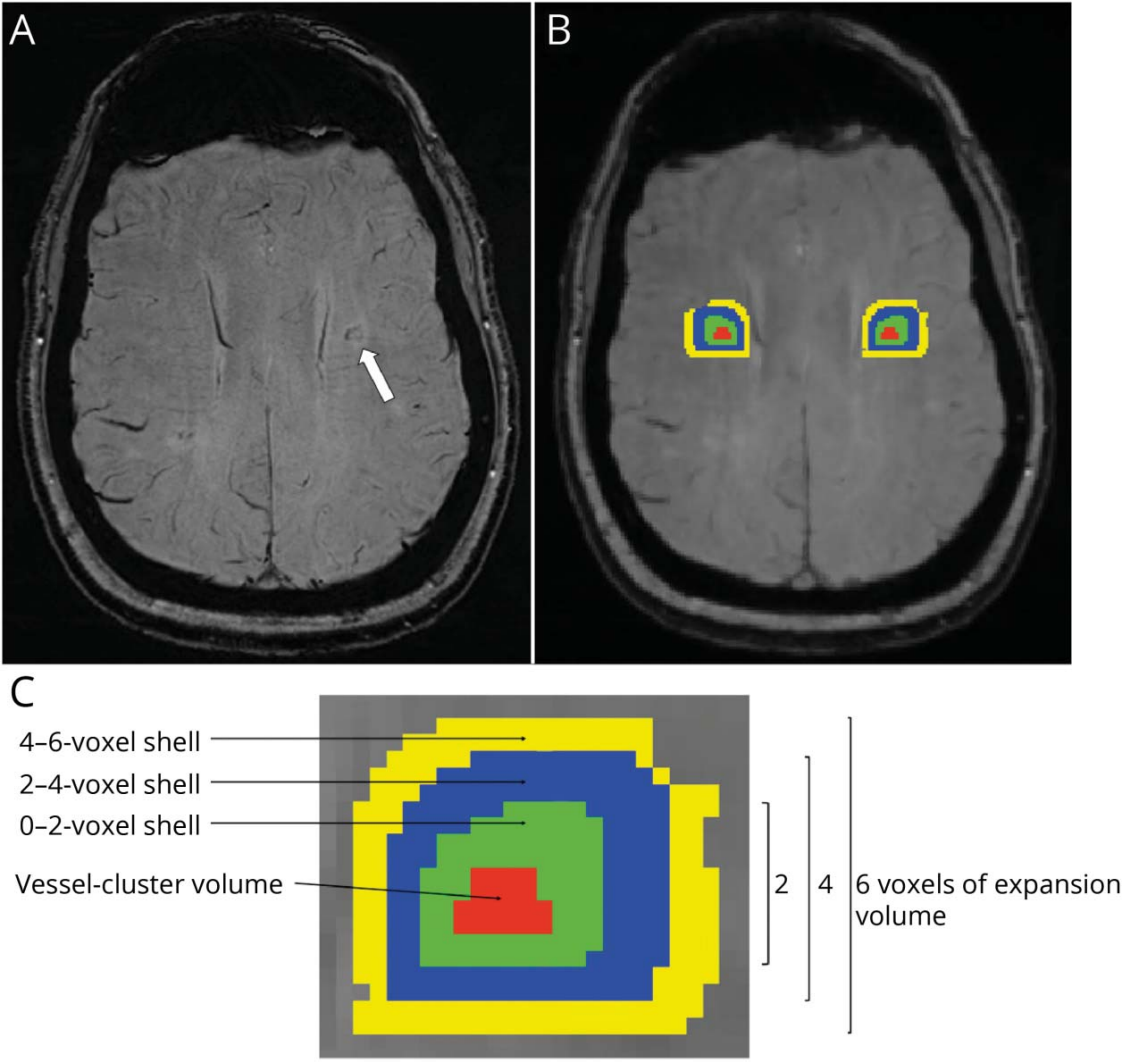
Vessel-Cluster Volume Masking and Processing of Additional Volume Expansions and Contralateral Corresponding Volumes (A) A vessel-cluster is indicated with a white arrow on susceptibility-weighted imaging (SWI) in the left centrum semiovale of a patient with CADASIL. (B) The volume covered by the vessel-cluster is shown in red on the image-processing viewer (FSL). Additional concentric shell volumes (at 0–2, 2–4, and 4–6 voxels from the vessel-cluster segmentation) represented using different colors (green, blue, and yellow, respectively), were generated in 3 dimensions within the white matter (C represents an augmentation of the area including the vessel-cluster volume segmentations). Contralateral-mirrored volumes (represented with the same colors) were generated in the contralateral hemisphere for the analysis. Notice that the resolution on SWI (A) was higher than on the nifti-file–converted image-processing viewer (FSL). The former was used for qualitative analysis, and the latter only to obtain quantitative measures.

Finally, to assess CVR magnitude and delay in the vessel-clusters and in the surrounding tissue, from the original vessel-cluster segmentations, we generated 3 additional concentric circumferential 3D expansions (shells) around the vessel-clusters, each 2 voxels thick in T2-w space (which approximates 1 voxel in CVR data) limited to the white matter, as presented in Figure 2. Then, we automatically generated contralateral-mirrored segments within the white matter, checked these for the accurate mirror-image location, and edited manually if required (E.C. and S.R.). FLS software^16^ was used for the mask processing.

### Statistical Analysis

Variables were described as mean and SD, median and IQR, or absolute and relative frequency as appropriate. Continuous and ordered variables were compared between patients with and without vessel-clusters in univariable analysis using Student *t* test or Mann-Whitney *U* test as appropriate, while a Pearson 2 test or Fisher exact test was used for categorical variables. Interrater agreement between the 2 readers for the vessel-cluster assessment was assessed with kappa statistics (details in eMethods, links.lww.com/WNL/C29). No missing values were detected among variables, except for systolic and diastolic blood pressure at the time of MRI that were not available in 6 of the 76 patients (8%), and CVR data were not usable in 7 of the 76 patients (9%).

In per-patient analysis, we analyzed the factors associated with the presence of vessel-clusters (1 or more vessel-clusters) in univariable analysis. Then, the relevant factors identified in the univariable analysis (*p* < 0.1) were assessed in multivariable logistic regression analysis adjusted for specific preselected clinically relevant variables (age, sex, log10-normalized WMH volume, number of lacunes) and other according to the *p* < 0.1 significance level in univariable analysis (alcohol use, type of SVD, microbleeds, total PVS score), excluding CVR values for not being available in 7 patients. Continuous variables without normal distribution (normalized WMH volume) were log10 transformed in the multivariable analysis. Collinearity according to variance inflation factor (VIF) was identified between visual SVD scale/quantitative measures and log10-normalized WMH volume/type of SVD. In the multivariable analysis, log10-normalized WMH volume and type of SVD were both maintained (VIF = 4.11 and VIF = 4.01, respectively) for the assessment of the effect of other variables, while log10-normalized WMH was excluded to assess the association between vessel-cluster presence and CADASIL type of SVD, and vice versa.

As a sensitive analysis, we analyzed the factors associated with the per-patient number of vessel-clusters using ordinal regression in univariable and multivariable analyses. To achieve comparable groups of the dependent variable, we collapsed the number of 4 or more vessel-clusters into 1 category.

In per-cluster analysis, we assessed CVR magnitude and CVR delay in the original and expanded volumes using contralateral segmentations as a reference, using the 2-sample Student *t* test. To analyze the potential gradient CVR effect from the vessel-cluster volume through the concentric shell expansions, we assessed the presence of linear, quadratic, cubic, or quartic trends between the CVR magnitude values using orthogonal polynomial contrasts in mixed model analysis.

We considered as statistically significant *p* < 0.05, and all hypotheses were 2-sided. All statistical analyses were performed using Stata/IC 15.1 for Mac (StataCorp, College Station, TX). Graphpad Prism 8.4 for Mac was used for graphing.

### Data Availability

Data not provided in the article because of space limitations may be shared (anonymized) at the reasonable request of any qualified investigator for purposes of replicating procedures and results.

## Results

Seventy-seven patients were recruited in the INVESTIGATE-SVD study from November 2017 to September 2019. Patients with sporadic SVD were recruited in Edinburgh (n = 25) and Maastricht (n = 20), and patients with CADASIL in Munich (n = 32). All patients completed the main structural sequences. However, SWI sequences from 1 patient with CADASIL were seriously affected by movement artifacts, and that patient was excluded from the analysis. The mean age (SD) of the selected patients was 57 years (12) (64 years [SD 11] in sporadic SVD and 53 years [11] in CADASIL patients), and 35 of the 76 patients (46%) were male (26 [58%] in sporadic SVD and 15 [48%] in CADASIL patients). Current smoking and alcohol use were frequent (53% and 71%, respectively). The most common vascular risk factors were history of hypertension and hyperlipidemia (61% and 59%, respectively). None of the patients had atrial fibrillation, and 80% of the patients were on antiplatelet treatment (no patients were on anticoagulant treatment). On MRI evaluation, the SVD structural features were more severe in patients with CADASIL than in those with sporadic SVD. Detailed information on clinical and radiologic features of the study cohort is summarized in Table 1.

**Table 1:**
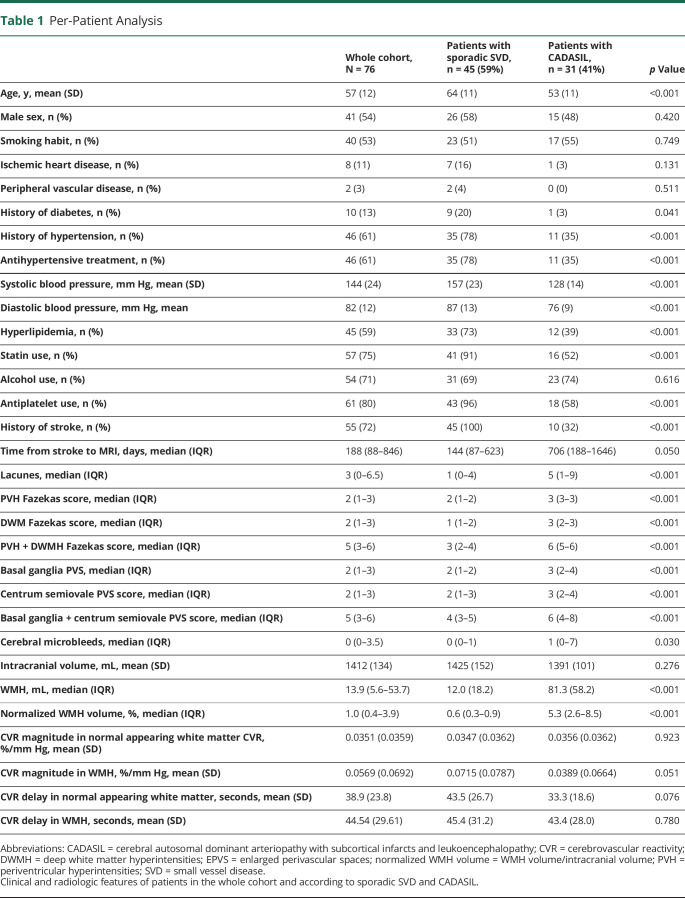
Per-Patient Analysis

### Per-Patient Analysis

**Figure 3:**
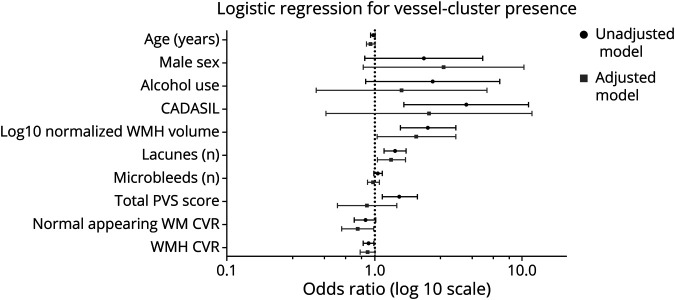
Multivariable Logistic Regression Analysis for the Presence of Vessel-Clusters Multivariable analysis was adjusted for age, sex, alcohol use, log10-normalized WMH volume, number of lacunes, type of SVD, microbleeds, and total PVS score. WM: white matter; WMH: white matter hyperintensities; CADASIL: cerebral autosomal dominant arteriopathy with subcortical infarcts and leukoencephalopathy; PVS: perivascular spaces.; CVR: cerebrovascular reactivity.

Thirty-six of the 76 patients (47%) showed at least 1 vessel-cluster (22 had more than 1 vessel-cluster, with almost symmetrical distribution, eFigure 4, links.lww.com/WNL/C29). In the univariable analysis, a diagnosis of CADASIL, alcohol use, and increased severity of SVD on structural imaging were associated with the presence of vessel-clusters, whereas no associations were found for age, sex, other vascular risk factors, or concomitant antithrombotic treatment (Table 2). In the multivariable analysis, CADASIL subtype and alcohol use were no longer associated with the presence of vessel-clusters, and among structural imaging variables, only the number of lacunes (OR = 1.30; 95% CI, 1.05–1.62; *p* = 0.018) and normalized log10 WMH volume value (per-log10 increase, OR = 1.92; 95% CI, 1.04–3.56; *p* = 0.038) remained significant (Figure 3). Among the 69 patients with usable CVR-derived measures, the presence of vessel-clusters was independently associated with lower CVR magnitude in the normal appearing white matter in multivariable analysis (per %/100-mm Hg, OR = 0.77, 95% CI, 0.60–0.99; *p* = 0.040) and a trend for lower CVR in WMH (per %/100-mm Hg OR = 0.90; 95% CI, 0.80–1.01; *p* = 0.069) (Figure 3). The detailed analysis is available in eTable 1.

**Table 2:**
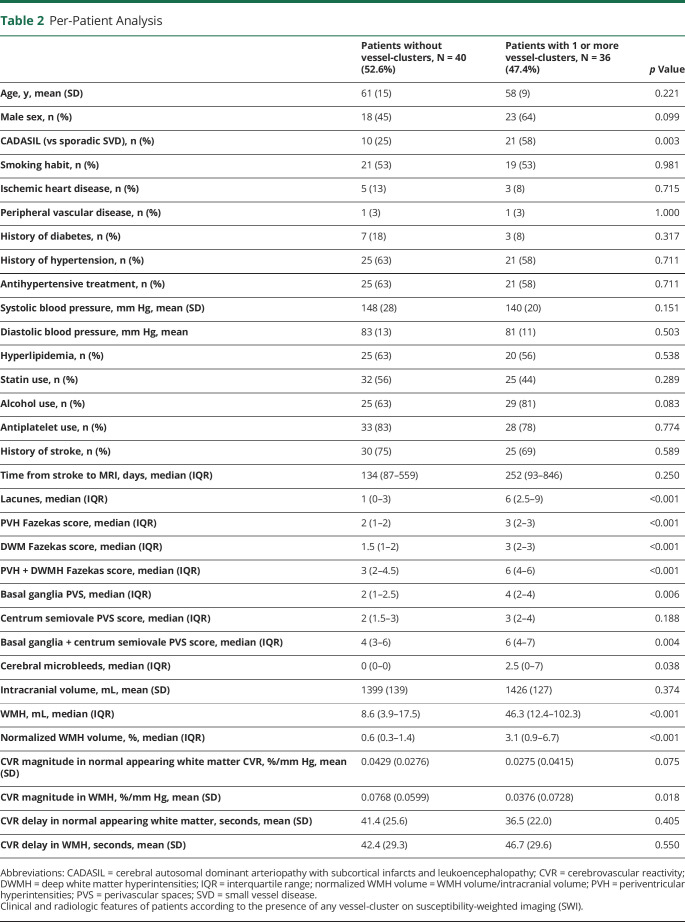
Per-Patient Analysis

In the ordinal regression analysis for the per-patient number of vessel-clusters (OR; 95% CI; *p* value), age (per year, 0.94; 0.88–0.99; 0.037), male sex (3.63; 95% CI; 1.29–10.24; 0.015), the number of lacunes (1.22; 1.03–5.31; 0.002), and normalized log10 WMH volume value (per-log10 increase, 1.80; 1.09–2.96; 0.021) remained significantly associated with the number of vessel-clusters after adjusting for covariates, whereas CVR (per %/100-mm Hg) in both normal appearing white matter (0.88; 0.77–1.01; 0.069) and WMH (0.93; 0.86–1.01; 0.088) had a nonsignificant negative trend (eTable 2, links.lww.com/WNL/C29).

### Per-Cluster Analysis

**Figure 4:**
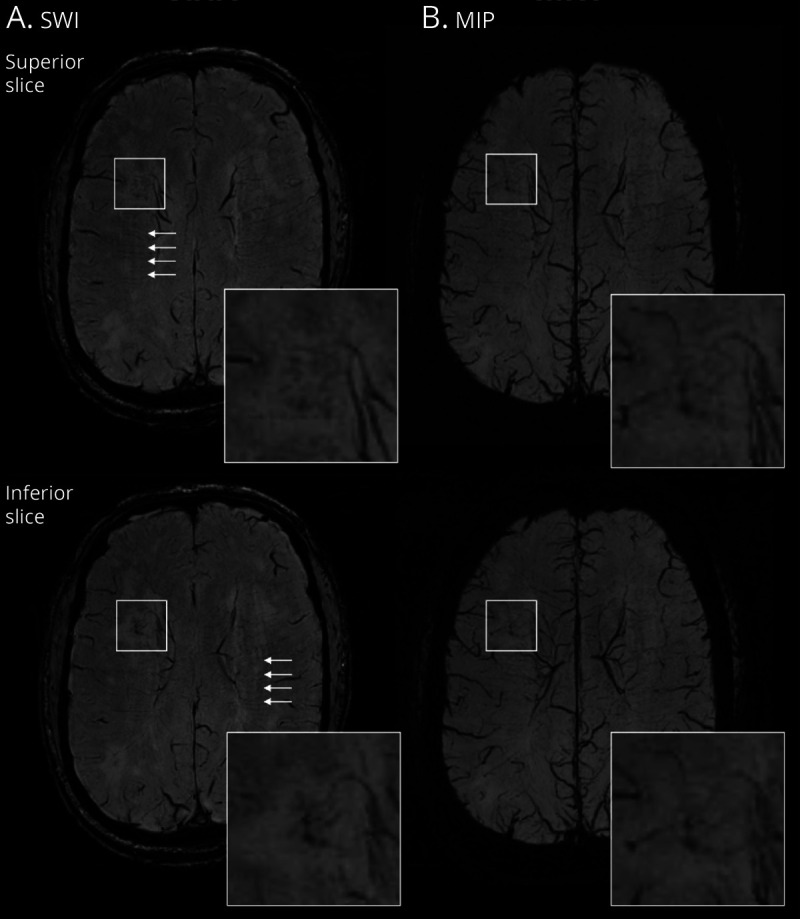
Example of a Vessel-Cluster Showing Vessels Draining to the Deep Venous System Representative vessel-cluster appearance in consecutive axial slices in a patient with sporadic small vessel disease. The vessel-cluster (square) is shown in 2 consecutive axial slices (superior and inferior) on susceptibility-weighted imaging (SWI, A) and maximum intensity projection (MIP, B) sequences. In the enhancements of the vessel-cluster regions, small vessels are visible as dot-like appearance that are traceable in the contiguous slice or in the MIP sequences enhancing the visibility of these structures through different planes. Notice that most of the vessels from the cluster converge to veins draining to the deep venous system, but the distribution of these vessels is disordered and different from the normal parallel appearance of the deep medullary venules visible in centrum semiovale (white arrows).

A total of 94 vessel-clusters were identified among 36 patients. Forty-eight of the 94 vessel-clusters (51%) were located in the left hemisphere, 22 of 94 (23%) were located in the anterior, 55 (58.5%) in the middle, and 17 (18%) in the posterior deep white matter. The median (IQR) volume of the region containing the vessel-cluster was 0.15 mL (0.08–0.26), which had a round shape in 45 (48%) vessel-clusters, ovoid in 32 (34%), irregular in 6 (6%), and linear in 11 (12%). Among the 94 vessel-clusters, 27 of them showed clustered or linked low-signal dots or lines that seem to correspond to 1 main vessel-like structure (29%), while clusters of multiple vessel-like structures (up to 5) were observed in the remaining 67 (71%), with a median of 2 (1–3) vessel-like structures per vessel-cluster (examples in Figure 1 and eFigure 5, links.lww.com/WNL/C29). An example of the traceability of vessel-like structures on SWI and MIP through contiguous axial planes is shown in Figure 4. Twelve vessel-clusters (13%) corresponded to noncavitated WMH, 45 (48%) to partial cavitation in WMH, and 37 (40%) to complete cavities. Vessel-clusters of multiple vessels covered a larger volume than single–vessel-like structures (median [IQR] volume [mL]: 0.17 [0.98–0.30] vs 0.09 [0.5–0.20], respectively, *p* = 0.005), were more likely to be associated with completely cavitated lesions (n [%], 33 [49] vs 4 [15], *p* = 0.002), were more likely to appear as a linear rim (n [%], 37 [44] vs 4 [15], *p* < 0.001), but with no difference in location in the white matter (*p* = 0.186). In the 12 vessel-clusters corresponding to noncavitated regions, we observed 1 main dilated vessel inside an area of WMH (eFigure 5, links.lww.com/WNL/C29), whereas vessel-clusters associated with partial and full cavitation (33% and 70%, respectively) had a linear low-signal rim appearance on SWI in the edges of a cavity (Figure 1). More details of structural vessel-cluster features are available in eTable 3.

**Figure 5:**
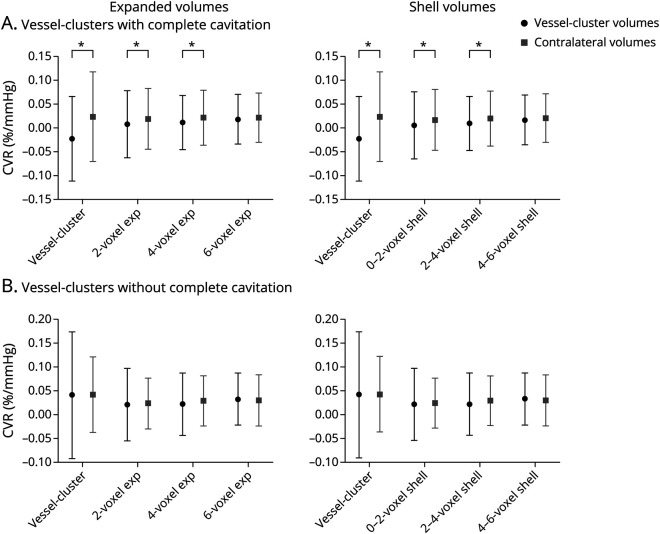
Per-Cluster Cerebrovascular Reactivity Magnitude Analysis Compared With Contralateral Volumes, According to the Presence or Absence of Complete Cavitation Forest plot analysis in the 36 vessel-clusters with complete cavitation (A) and in the 57 vessel-clusters without complete cavitation (B). Black points (vessel-cluster volumes) and gray squares (contralateral volumes) represent mean cerebrovascular reactivity (CVR), and bars represent SD. *Represents a level of significance (*p*) in the 2-sample *t* test <0.05. On the left side of the panel, the charts represent CVR within concentric expansions of the vessel-clusters (including it), while on the right side of the panel, the charts represent CVR within the original cluster and concentric shells independently.

For each vessel-cluster (per-cluster analysis), we assessed CVR magnitude in the vessel-cluster volume and in concentric shells in the surrounding tissue. CVR magnitude from the vessel-cluster volumes was available in 73 of 94 (76%) after excluding small volumes that were poorly coregistered with CVR maps, and expanded volumes and penumbral shells were available (93/94 patients, 99%). In vessel-cluster volumes (including volumes up to 4-voxel expansion) corresponding to complete cavities, the CVR magnitude was lower compared with the contralateral volumes (2-sample Student *t* test). The white matter surrounding these vessel-clusters (up to 4-voxel shells) also had lower CVR compared with mirrored volumes. These differences in CVR magnitude were not significant in vessel-cluster volumes corresponding to partial or no cavitation (Figure 5, eTable 4, links.lww.com/WNL/C29).

Finally, in the group of vessel-clusters with complete cavitation, there was a significant linear gradient for increasing CVR magnitude values from the vessel-cluster volume through concentric penumbral shells (contrast 0.015, 95% CI 0.007–0.023, z = 3.91, *p* < 0.001), but this gradient was not present in clusters without full cavities (contrast −0.002, 95% CI –0.008 to 0.005, z = −0.49, *p* = 0.624), interaction *p* < 0.001 (eFigure 6, links.lww.com/WNL/C29).

The per-cluster analysis assessing mean CVR delay difference between vessel-cluster volumes or surrounding tissue and contralateral volumes did not show any significant difference, as detailed in the eFigure 7, links.lww.com/WNL/C29.

## Discussion

We describe what appears to be a new feature on SWI sequences in patients with severe SVD consistent with small clusters of dilated small vessels in WMH and associated with the formation of cavitation. The vessel-clusters were more likely to be seen in patients with large WMH volume and were highly associated with both the overall number of lacunes and colocated with what appeared to be cavities at different stages of formation. The prevalence of vessel-clusters in patients with CADASIL was higher compared with patients with sporadic SVD, but the association disappeared after adjustment for age, severity of SVD, and other SVD-related risk factors. The association with reduced CVR in white matter surrounding cavities suggests that the vessel-clusters represent dysfunctional and dilated small vessels. Therefore, vessel-clusters on SWI seem to be a feature associated with severe “cavitating” SVD that may be observed in patients with either sporadic or genetic SVD and may indicate that the small deep vessels are in an advanced stage of exhaustion of their ability to vasodilate resulting in worsening tissue damage.

The identification of the vessel-clusters on SWI is based on the assumption that small parenchymal vessels may be visualized as low signal (dark) if they contain deoxygenated hemoglobin due to the latter's paramagnetic distortion of the magnetic field.^17^ In this way, typically, normal small deep cerebral venules can be visualized on SWI.^5-8^ In this study, we described focal alterations on SWI appearing as small abnormal dilated vessels associated with focal white matter degeneration in SVD. It is possible that other paramagnetic elements may produce a dark signal on SWI in the brain in SVD. Small amounts of hemosiderin are commonly present in or at the edges of lacunes in the brain representing petechial hemorrhage as part of the natural history of ischemic stroke,^18^ which would be consistent with the rim-like distribution on the wall of complete cavities in some cases of our cohort (Figure 1C). However, it is also possible that residual vessels may persist on the edges of lacunes. Small platelet-erythrocyte aggregates containing deoxygenated hemoglobin could also produce similar artifacts on gradient-echo–based imaging, as described in small subcortical infarcts,^19,20^ and it is conceivable that local thrombosis could be triggered by slow blood flow and endothelial dysfunction. Tissue interfaces may also cause magnetic field effects (i.e., between a cavity and tissue) but are likely to be more subtle than those identified as vessel-clusters (eFigure 3, A, D, links.lww.com/WNL/C29). Finally, the SWI signal may overestimate the true size of a paramagnetic structure due to blooming artefact, meaning that the actual size of the vessel-like structures in the SWI vessel-clusters may be much smaller than depicted on MRI.^4^ Nevertheless, the traceable appearance of small tubular structures through contiguous axial planes, some of them finally draining to the deep venous system (Figure 4), makes these findings hard to be interpreted with an alternative hypothesis other than dilated disorganized small vessels, possibly originally arterioles or venules or something in between, corresponding to white matter injury.

The results of this study should not be extrapolated to images obtained using 1.5 Tesla MRI, but it is likely that at least the more apparent vessel-clusters might be visible similarly to deep medullary veins despite lower resolution compared with 3 Tesla MRI.^21^ However, further studies are needed to confirm whether and to what extent vessel-cluster may be assessed on 1.5 Tesla SWI-MRI. Low-definition MRI or the use of other gradient-echo–based techniques could in part be the reason why these findings have not been described before or might have been overlooked or considered as tortuosity of the deep medullary veins or hemorrhagic features in SVD.

In per-patient multivariable analysis, we observed a weak relationship between decreased CVR in the white matter (both normal appearing white matter and WMH) and the presence of vessel-clusters. However, changes in CVR are subtle, and the influence of different factors might not be easy to control in global analysis.^22^ For this reason, we assessed the values of CVR measures in per-cluster analysis, using the corresponding volume in contralateral brain white matter area as a reference. This showed that CVR magnitude depended on the degree of cavitation: In the subgroup of vessel-clusters with complete cavitation, as expected, CVR in the vessel-cluster volume was lower than contralateral white matter due to CSF content. However, the surrounding white matter up to 4-voxel concentric shells also had lower CVR than the corresponding contralateral volumes. The presence of a linear gradient increasing centrifugally from the vessel-cluster through the penumbral shells confirmed that CVR was impaired around cavities with vessel-clusters. In clusters with incomplete cavitation, we did not find reductions in CVR across the volume. These results suggest that CVR is reduced in the tissue surrounding cavities because of exhausted vasodilatory reserve, while the visibility on SWI indicates that they contain deoxyhemoglobin resulting from maximum oxygen extraction,^23,24^ representing concurrent factors contributing to the blooming effect on SWI enabling the cluster visualization. On the other hand, it is possible that the noncavitated and early-cavitated vessel-clusters still maintain CVR but are approaching a stage where vascular function fails. Hence, the results from the CVR analysis have to be interpreted cautiously because of the cross-sectional design, the small sample size and variability of vessel-clusters in size, and corresponding tissue characteristics and require confirmation in longitudinal and larger cohorts.

Vessel-clusters were associated with a range of stages of cavity formation in the white matter, adding further complexity to the theories of lacune formation. It is generally believed that most lacunes are the result of ischemic necrosis after infarction due to a perforating arteriolar occlusion.^25^ However, incident lacunes in CADASIL predominantly appear on the edges of WMH rather than following a distal vascular distribution, thus supporting the concept of alternative mechanisms.^26^ Lacunes have also been related to progressive pathologic changes in deep venule walls in venous collagenosis.^27,28^ As suggested in the current analysis, microvascular dysfunction could lead to gradual tissue damage ending in lacune formation that could be less symptomatic due to its gradual occurrence than in acute infarctions.^29,30^ In this scenario, vessel-clusters might represent over-dilated vessels (i.e., postcapillary venules) that cannot respond further to vasoactive stimuli in regions showing tissue degeneration. Unfortunately, the cross-sectional design of our study precludes the assessment of causal or longitudinal relationships between the vessel-clusters and white matter cavitation. However, the association of vessel-clusters with low CVR is consistent with focal loss of homeostasis and worthy of future longitudinal studies to determine the pathophysiologic significance and hypothetical role in cavitation processes leading to lacune formation.

The main associations of some structural radiologic SVD markers such as lacunes and WMH with vessel-cluster presence were confirmed in ordinal regression analysis for the number of vessel-clusters, while CVR magnitude was no longer significant but still showed a negative trend. Interestingly, we observed a significant association between male sex and the number of vessel-clusters, which might reflect different severity and SVD patterns depending on specific genetic and hormonal profiles. However, the differences in SVD radiologic and clinical manifestations attributable to sex are still largely unknown.^31^

The homogeneity of the imaging acquisition and processing across all enrolling centers represent major strengths of this study. This study also included patients with sporadic and genetic SVD, which allowed us to assess the new feature on SWI in patients believed to have different SVD mechanisms.

This study also has some limitations. First, the size sample might not have been sufficient to detect subtle differences in vascular function measures. However, the patients of this cohort presented moderate-to-severe SVD, thus mitigating the effect of the small sample and increasing the likelihood of SVD-related findings. Second, the interrater agreement was “good,” not excellent, and external validation in independent cohorts is warranted. Third, the nature of the vessel-clusters is open to many interpretations, some of them discussed above. However, the main aim of this study was to describe the prevalence, associations, and characteristics of this new radiologic sign. Further correlates including on pathology can now be examined in further studies. Fourth, the limited z definition (3 mm) of the SWI sequences precluded a 3-dimensional analysis of the findings, and the spatial resolution of the CVR BOLD sequence that limits precise geographical localization and values within the cavitated vessel-clusters may have been affected by CSF in cavities. However, CVR magnitude values were also lower in the tissue surrounding cavities, demonstrating the impairment in vascular reserve in these penumbral areas. Finally, the presence of vessel-clusters has not been assessed in a control group without SVD. However, the likelihood of finding vessel-clusters in brains without small vessel lesions is expected to be extremely low because none of the vessel-clusters in our series corresponded to normal appearing white matter, their presence was highly dependent on burden of lacunes and WMH, and furthermore, we have not observed similar findings on SWI in any of our healthy research volunteers over many years of MRI scanning.

Vessel-clusters may be observed in up to half of the patients with moderate-to-severe SVD, including both sporadic and genetic types. The vessel-clusters are related to damaged tissue showing different grades of cavitation in WMH. The properties of deoxygenated blood on susceptibility-weighted sequences and the association with lower CVR in the white matter and in the surrounding tissue in clusters with cavities suggest that the vessel-clusters represent maximal of small deep vessels and oxygen extraction in white matter that is approaching terminal injury and cavitation. The pathophysiologic significance of this new feature warrants confirmation in imaging techniques with higher resolution (i.e., 7 Tesla MRI), pathology studies, and further longitudinal investigation that should assess whether vessel-clusters predict cavitation in SVD and even contribute to lacune formation, or are an epiphenomenon of white matter injury.

## Glossary

BOLD: blood-oxygen-level-dependent
CADASIL: cerebral autosomal dominant arteriopathy with subcortical infarcts and leukoencephalopathy
CVR: cerebrovascular reactivity
FLAIR: fluid-attenuated inversion recovery
IQR: interquartile range
MIP: maximum intensity projection
PVS: perivascular spaces
SVD: small vessel disease
SWI: susceptibility-weighted imaging
VIF: variance inflation factor
WMH: white matter hyperintensity
